# Relative Validity of a Beverage Frequency Questionnaire Used to Assess Fluid Intake in the Autosomal Dominant Polycystic Kidney Disease Population

**DOI:** 10.3390/nu10081051

**Published:** 2018-08-09

**Authors:** Carly Mannix, Anna Rangan, Annette Wong, Jennifer Zhang, Margaret Allman-Farinelli, Gopala Rangan

**Affiliations:** 1 Centre for Transplant and Renal Research, The Westmead Institute for Medical Research, The University of Sydney, Westmead, NSW 2145, Australia; annette.wong@sydney.edu.au (A.W.); jennifer.zhang@sydney.edu.au (J.Z.); g.rangan@sydney.edu.au (G.R.); 2 Department of Renal Medicine, Westmead Hospital, Western Sydney Local Health District, Westmead, NSW 2145, Australia; 3 School of Life and Environmental Sciences, The Charles Perkins Centre, The University of Sydney, Sydney, NSW 2006, Australia; anna.rangan@sydney.edu.au (A.R.); margaret.allman-farinelli@sydney.edu.au (M.A.-F.)

**Keywords:** fluid intake, hydration, kidney, polycystic kidney disease, questionnaire

## Abstract

Maintaining hydration sufficient to reduce levels of arginine vasopressin has been hypothesised to slow kidney cyst growth in autosomal dominant polycystic kidney disease (ADPKD). The semi-quantitative beverage frequency questionnaire (BFQ) was designed to measure usual fluid intake over the past month. The aim of this study was to assess the validity and reliability of the BFQ compared with the 24-h urine biomarkers. Participants with ADPKD (18–67 years; estimated glomerular filtration rate (eGFR) ≥ 30 mL/min1.73 m^2^) completed the BFQ. Serum creatinine, eGFR, 24-h urine volume, and osmolality were measured. Pearson correlation coefficients, paired *t* test, and Bland–Altman plots were used to evaluate agreement between the methods. A subset repeated the BFQ to assess reliability. A total of 121 participants (54% male, 43 ± 11 years; mean ± SD) completed the BFQ and at least one 24-h urine collection. The correlation between the BFQ and the 24-h urine volume was moderate (*r* = 0.580) and weaker with the 24-h urine osmolality (*r* = −0.276). The Bland–Altman plots revealed good agreement between the BFQ and the 24-h urine volume with no obvious bias; however, the limits of agreement were wide (−1517–1943 mL). The BFQ1 and BFQ2 were strongly correlated (*r* = 0.799, *p* < 0.001) and were not significantly different (*p* = 0.598). The BFQ is a valid and reliable tool to assess the usual fluid intake of the ADPKD population.

## 1. Introduction

Autosomal dominant polycystic kidney disease (ADPKD) is the most prevalent genetic kidney disease in adults, with an incidence of one in every 2500 individuals, and is the cause of kidney failure in ~5–10% of the dialysis population worldwide [1]. It is due to mutations in either of the polycystic kidney disease (PKD) genes (PKD1 or PKD2), which leads to the progressive formation of multiple fluid-filled cysts in the kidneys during life and causes kidney failure by the age of 60 in half of the affected patients [1]. At present, there is no treatment that can cure ADPKD or prevent the formation of cysts in the kidneys.

Arginine vasopressin (AVP) is a hormone responsible for regulating water homeostasis [2] and is the single most important endogenous factor potentiating cyst growth in ADPKD during the postnatal period [3]. As serum osmolality increases, AVP is released, generating cyclic 3′5-monophosphate (cAMP), resulting in water reabsorption in the kidney. As increased fluid intake decreases the release of AVP, it has been hypothesised that it may slow the growth of renal cysts in ADPKD [4,5]. 

To assess and monitor the fluid intake of patients with ADPKD, a validated tool that is easy and time efficient to administer in population-based studies is required. From the current literature and implemented practices, there are no validated tools to assess the fluid intake of patients with ADPKD. Therefore, this study adapted the previously validated semi-quantitative self-administered beverage intake questionnaire (BEVQ), which quantified habitual beverage intake in the general population [6], to construct the beverage frequency questionnaire (BFQ). The BFQ includes a visual portion size guide to assist participants with volume estimation. 

In validation studies, biomarkers are likely to demonstrate greater accuracy as reference standards compared to dietary assessment methods, because they are objective, do not rely on memory, and are not subject to measurement error, such as over- or underreporting [7,8]. However, there is no consensus on the ‘ideal’ biomarker that accurately reflects fluctuations in hydration status in response to fluids intake, because each method is a compromise between practical limitations and accuracy [8,9,10]. In previous studies, strong correlations were found between the fluid intake, 24-h urine volume, and osmolality, and for this reason, they are the preferred method for evaluating daily water balance [11]. Therefore, the aim of this study was to assess the relative validity of the BFQ compared with the 24-h urine volume and osmolality, and its reliability using the test–retest method.

## 2. Materials and Methods 

### 2.1. Human Research Ethics Approval

This project is a sub-study of the PREVENT-ADPKD trial [12], registered with Australian New Zealand Clinical Trials Registry (ANZCTR, No. ACTRN12614001216606). The study was conducted according to the Declaration of Helsinki and approved by the Western Sydney Local Health District (WSLHD) Human Research Ethics Committee (HREC), HREC ref: AU RED HREC/14/WMEAD/414. 

### 2.2. Study Population 

A total of 121 participants recruited in the PREVENT-ADPKD trial from December 2015 to August 2017 were included in this study. Written informed consent was obtained from all the participants. Participant eligibility criteria have been previously published [12]. Briefly, the participants were screened from a retrospective review of clinic notes or by referral by a nephrologist using the following criteria: 18–67 years of age; diagnosed with ADPKD according to the Pei–Ravine unified ultrasound criteria and an estimated glomerular filtration rate (eGFR) ≥30 mL/min/1.73 m^2^. Participants who presented a safety risk; had a contraindication for magnetic resonance imaging (MRI) assessment; were judged to be non-compliant; had a confounding illness or treatment, and/or were currently participating in another clinical trial were excluded from the study. 

### 2.3. Study Design

**Figure 1:**
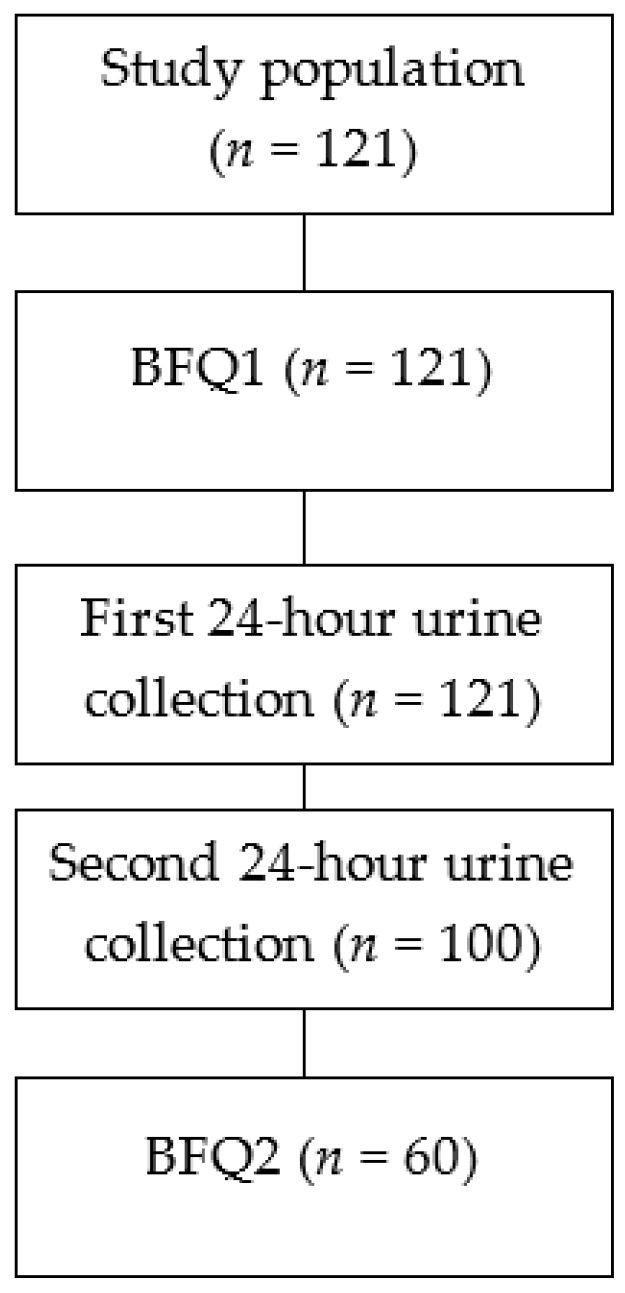
Flow chart of the stages of the study. BFQ: beverage frequency questionnaire.

At the initial visit, height, body weight, and body mass index (BMI) were determined. Three blood pressures were measured, and the average was used for analysis. All the participants completed the semi-quantitative BFQ with the assistance of a dietitian and were instructed to complete two 24-h urine collections and blood tests within 12 weeks of the visit date. Urine volume and osmolality were measured from the 24-h urine collections by an external pathology laboratory. From the blood test, serum creatinine was measured, and eGFR was determined by the Chronic Kidney Disease Epidemiology Collaboration (CKD-EPI) formula. The participants also completed a standardised renal MRI to assess height adjusted total kidney volume (ht-TKV). The analysis of TKV was conducted by personnel at the Translational PKD Center at Mayo Clinic, Rochester, Minnesota (United States of America, USA), using de-identified MR images [13]. Once participants completed two 24-h urine collections, blood tests, and the baseline MRI scan, they were randomised (1:1) using a computer-based tool (www.randomize.net) into either the control (usual fluid intake) or the intervention (prescribed fluid intake) group according to the design of the PREVENT-ADPKD Study [12]. The participants randomised into the intervention group repeated the BFQ prior to receiving the intervention of the study. Figure 1 summarises the stages of the present study. 

### 2.4. Development of the Beverage Frequency Questionnaire (BFQ)

The BFQ is a semi-quantitative food frequency questionnaire that was developed based on the self-administered beverage intake questionnaire (BEVQ) by Hedrick et al. [6]. The adaptations were made by the study dietitians (C.M., A.W., and A.R.) to improve its applicability to the Australian population (e.g., including all commonly consumed beverage types and typical portion sizes; using Australian vernacular, such as changing “soda” to “soft drink”). To do this, the results of the 2011–2012 National Nutrition and Physical Activity Survey (NNPAS 2011–12) were analysed to identify beverages and portion sizes commonly consumed by the Australian population [14]. The BFQ consists of 14 beverage categories and two additional “other” categories for beverages not listed. The categories were created based on beverages with similar characteristics (energy and nutrient content). The BFQ utilises closed questions with eight options to determine consumption frequency, ranging from “never” to “4 + times per day”, along with four options to quantify the volume consumed, ranging from “0.5 cup or less” to “2 or more cups”. An open-ended question regarding total water intake over a 24-h day was used for water measurement. A visual portion size guide was included to assist participants with volume estimation. A copy of the BFQ and visual portion size guide are provided in Appendix A and Appendix B, respectively.

### 2.5. Calculation of Fluid Intake Using BFQ Responses

The total daily fluid intake for each participant was calculated as the sum of the daily intake of all beverages on the BFQ. To calculate the average daily fluid intake of each beverage, weekly frequencies were converted to daily frequencies by dividing by seven. Daily frequencies (“How often”) were then multiplied by the millilitres per selected portion size (“How much”).

### 2.6. Statistical Analysis

The total daily fluid intake, calculated from the BFQ, was compared to the total volume and osmolality of the first and second 24-h urine collections, as well as the mean of the two urine collections. A paired-samples *t* test was performed to determine if there was a statistically significant difference between the total daily fluid intake and 24-h urine volume. Pearson’s correlation coefficients were calculated and interpreted using Hinkle et al.’s reference values [15] to measure correlations between the total daily fluid intake, 24-h urine volume, and osmolality. The Bland–Altman method [16] was used to assess the agreement between the total daily fluid intake from the BFQ and 24-h urine volume. Bland–Altman plots were constructed with reference lines to indicate the mean difference and 95% limits of agreement (LOA; LOA = mean difference ± (1.96 × standard deviation)). The test–retest method was implemented to assess the reliability of the BFQ, with the Pearson’s correlation coefficient calculated to measure correlations between BFQ1 and BFQ2. Statistical analyses were performed using SPSS (IBM SPSS Statistics 24; Chicago, IL, USA). A *p*-value < 0.05 was considered statistically significant.

## 3. Results

### 3.1. Clinical Characteristics of the Study Population

A total of 121 adults (consisting of 65 men) participated in the validation study, of whom all completed the BFQ and the first 24-h urine collection, and 100 completed two 24-h urine collections. The first and second 24-h urine collections were completed a mean 27 ± 27 and 41 ± 28 days after the screening visit, respectively. Table 1 shows the mean baseline characteristics of the participants in this study. According to BMI, 64% of the participants were classified as overweight/obese, and the participants mostly identified themselves as Caucasian (70%), followed by Asian (21%), or other (9%).

### 3.2. Relative Validity of the BFQ

Table 2 shows the mean value and correlation coefficient for the following: (i) the total daily fluid intake from the BFQ; (ii) the 24-h urine volume; and (iii) the 24-h urine osmolality from each of the 24-h urine collections. The ranges for the BFQ (938–5518 mL), 24-h urine volume (1015–6275 mL), and osmolality (132–880 mOsm/kg) were wide. A moderate correlation of *r* = 0.580 was observed between the BFQ and the mean 24-h urine volume (*p* < 0.001). An inverse relationship was found between the BFQ and the 24-h mean urine osmolality, but the correlation coefficient of *r* = −0.276 was weaker (*p* = 0.002).

**Figure 2:**
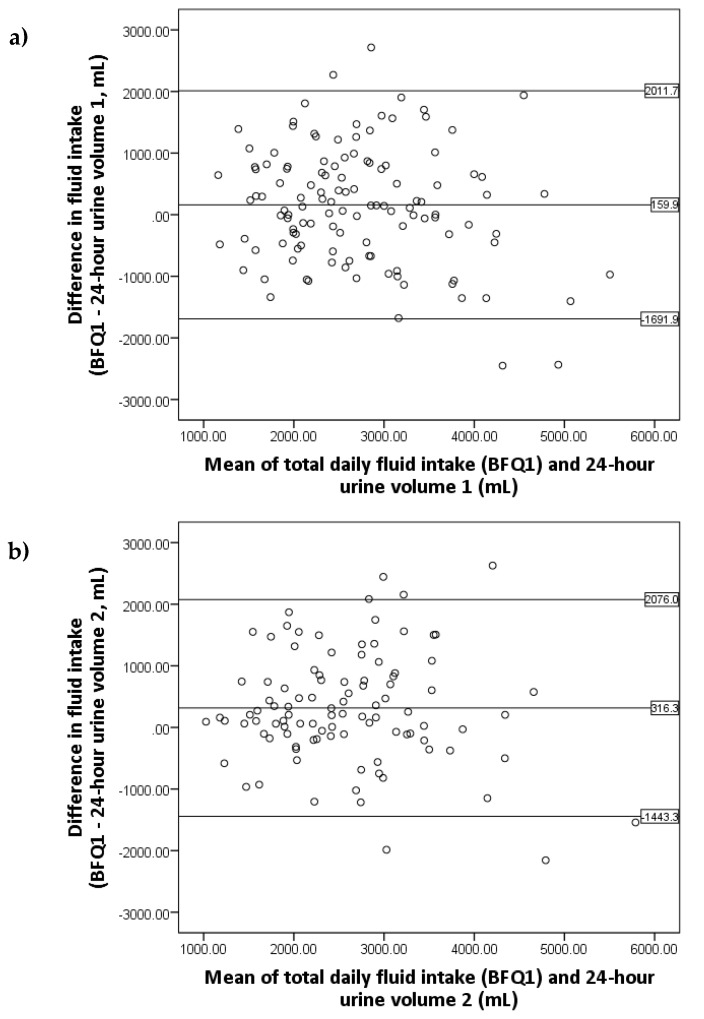

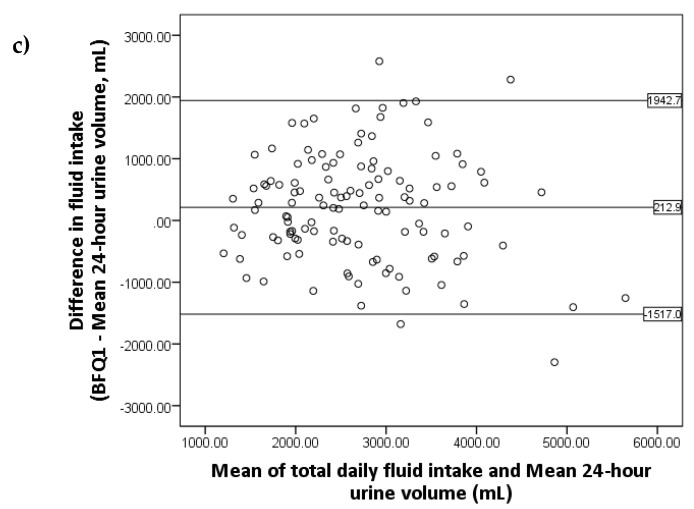
Bland–Altman plots of total daily fluid intake and (**a**) First 24-h urine volume, (**b**) Second 24-h urine volume, and (**c**) Mean 24-h urine volume with 95% limits of agreement.

The Bland–Altman plots showed good agreement between the BFQ and the 24-h urine volume (Figure 2). Data points were randomly scattered with no evidence of systematic bias; however, there were wide LOA between the two methods. A similar number of outliers can be seen above and below the LOA. A small but significant difference of 213 ± 883 mL was found between the BFQ and the mean 24-h urine volume (*p* = 0.009); however, this is not perceived as clinically significant, as urine output is likely lower due to insensible fluid loss.

### 3.3. Reliability of the BFQ

A total of 60 participants randomised to the intervention group of the PREVENT-ADPKD Study repeated the BFQ prior to receiving the intervention of the study. There was a mean of 83 ± 39 days between the completion of BFQ1 and BFQ2. Table 3 presents the mean values and correlation coefficient for BFQ1 and BFQ2. No significant difference was found between BFQ1 and BFQ2 (*p* = 0.598). A strong correlation was shown between BFQ1 and BFQ2 (*r* = 0.799, *p* < 0.001).

## 4. Discussion

Despite evidence indicating a potential relationship between levels of arginine vasopressin and renal cyst growth in ADPKD, no simple clinical tools to assess fluid intake in this population are available. In the present study, we developed the BFQ and validated its performance with the gold-standard of fluid intake, 24-h urine volume and osmolality, and also assessed its reliability in an adult ADPKD population. Our results indicated that the fluid intake, as determined by the BFQ, had a moderate positive correlation with the 24-h urine volume and a weaker inverse association with the 24-h urine osmolality. Moreover, the BFQ was reproducible when it was repeated in a subset of the study population. 

To our knowledge, no previous studies have validated a beverage intake questionnaire in a chronic kidney disease population. In fact, to date, we identified only three beverage questionnaire validation studies, and these were evaluated in a general healthy population [6,17,18]. Hedrick et al. [6] validated a questionnaire against food records (*r* = 0.456, *p* < 0.001); whereas, questionnaires by Ferreira-Pago et al. [17] and Malisova et al. [18] were validated against 24-h urine volume (*r* = 0.447, *p* = 0.0003; *r* = 0.29, *p* = 0.015, respectively) and osmolality (*r* = −0.447, *p* = 0.0005; *r* = −0.30, *p* = 0.010, respectively). In general, the correlations between the beverage questionnaires and the 24-h urine volume and osmolality in these previous studies were moderate to weak, are similar to the results of the current study (*r* = 0.580 and *r* = −0.276, all *p* < 0.05), and could possibly be due to the beverage questionnaire and the 24-h urine collections being completed on different days. The weaker correlations between the beverage questionnaires and the 24-h urine osmolality may also be due to the effect of dietary solute intake on urine osmolality [19]. In contrast, stronger correlations were found in a study [7], which completed electronic food and fluid diaries and samples of 24-h urine volume and osmolality on the same day (*r* = 0.74 to 0.79; *r* = −0.66 to −0.74, *p* < 0.001, respectively), likely due to reduced intra-individual variability. Additionally, in the study by Malisova et al. [18], moisture from food, estimated to account for 16.8% of total water intake, was included, which may provide a more accurate reflection of actual fluid intake.

In the present study, we did not evaluate the validity of the BFQ with other urinary hydration biomarkers such as specific gravity, pH, and colour, because previous data yielded conflicting results when these methods were used as reference standards. For example, in the study by Malisova et al., urine colour (*r* = −0.28, *p* = 0.033) correlated with the questionnaire, but no association was reported with urine specific gravity (*r* = −0.107, *p* = 0.403) and pH (*r* = −0.093, *p* = 0.483) [18]. In contrast, Hedrick et al.’s BEVQ inversely correlated with urine specific gravity on both administrations of the questionnaire (*r* = −0.202 and *r* = −0.238, respectively; *p* < 0.05) [6]. The reason for these conflicting results is not clear but suggests that the use of these biomarkers for the purpose of validation studies is probably not recommended. 

In the current study, a strong correlation was observed between BFQ1 and BFQ2 (*r* = 0.799, *p* < 0.001), with a mean interval of 83 days, suggesting the BFQ measured fluid intake consistently over time. This is similar to the reliability of the questionnaires by Hedrick et al. [6], Ferreira-Pago et al. [17], and Malisova et al. [18]. These studies also had variable intervals between questionnaire administrations ranging from 2 weeks to 12 months, with no significant difference in fluid intake estimation between the first and subsequent administrations. 

The BFQ tended to overestimate fluid intake by 213 mL, but this difference may have been a result of daily fluid imbalance. At the ADPKD population level, the Bland–Altman plots showed good agreement with data points distributed within two standard deviations of the mean and no obvious bias. However, the wide LOAs propose large variability in the reported fluid intake at the individual level. Therefore, the BFQ is likely to be a useful tool when implemented in population-based studies rather than in the screening of fluid intake of individual patients with ADPKD. In this regard, one potential translational application of the BFQ is its use in future randomised controlled trials, where it could be used to estimate intergroup differences in the baseline fluid intake of an ADPKD cohort prior to an intervention. With regard to the latter, a key advantage of the BFQ is that the questionnaire is simple and quick to administer.

One of the major strengths of this study was the use of objective urine biomarkers (24-h urine volume and osmolality) instead of dietary assessment methods, as they avoid bias caused by measurement errors, as discussed earlier. Furthermore, the use of two 24-h urine collections allowed for better representation of usual urine output, as a single 24-h urine collection may not be accurate due to intra-individual variance. Additionally, fluid intake questionnaires can provide a better indication of habitual fluid intake at the population level compared with a single 24-h recall, as beverages consumed episodically may not be reflected in the latter. 

A limitation of the present study is the semi-quantitative nature of the BFQ, as participants are confined to the frequency and quantity options specified, which may not accurately reflect their fluid intake. Likewise, some options provide a range (e.g., “4–6 times per week”), which does not distinguish the participant’s exact fluid intake. There is also potential for over- or underreporting, as the BFQ measures retrospective fluid consumption (not including moisture from food), which relies on the participant’s memory and their ability to accurately estimate portion sizes. Additionally, the participants were not instructed to complete the 24-h urine collections on a work and off-work day. Lastly, insensible fluid loss (due to metabolism; respiratory, skin, and gastrointestinal losses; and variations associated with season) was not specifically assessed in this study due to the complexity of obtaining accurate measures [20].

In conclusion, the results of this study revealed good agreement between the BFQ and the 24-h urine biomarkers, demonstrating that the BFQ is a relatively valid and reliable tool for assessing the fluid intake of the ADPKD population. Hence, the BFQ could be implemented in population-based studies where time-consuming and resource-intensive dietary assessment methods are not suitable. In addition, as the current study comprised of only participants with diagnosed ADPKD, the external validity of these results is limited to this population. However, our data provide the rationale for the future evaluation of the BFQ in other patient cohorts of chronic kidney diseases.

## Figures and Tables

**Table 1:** Baseline characteristics of the study population (*n* = 121).

Characteristics	Mean	SD	Minimum	Maximum
Age (years)	43	11	19	66
Height (cm)	171	11	146	195
Mass (kg)	80	19	51	137
BMI (kg/m^2^)	27	5	19	42
Systolic BP (mmHg)	135	14	98	172
Diastolic BP (mmHg)	83	10	62	111
eGFR (mL/min/1.73 m^2^)	73	19	25	90
Creatinine (µmol/L)	100	38	58	235
Ht-TKV (mL/m)	902	673	156	4504

SD, standard deviation; BMI, body mass index; BP, blood pressure; eGFR, estimated glomerular filtration rate; and Ht-TKV, height-corrected total kidney volume.

**Table 2:** Total daily fluid intake as measured by the beverage frequency questionnaire at screening (BFQ1), 24-h urine volume, and 24-h urine osmolality.

	*n*	Mean	SD	Minimum	Maximum	*p* *	*r*
BFQ1 (mL)	121	2797	919	938	5518	N/A	N/A
Urine volume 1 (mL)	121	2637	1075	690	6150	0.065	0.561 †
Urine volume 2 (mL)	100	2435	1006	770	6560	0.001	0.576 †
Mean urine volume (mL)		2584	1000	1015	6275	0.009	0.580 †
Urine osmolality 1 (mOsm/kg)	120	421	183	123	923	N/A	−0.283 ‡
Urine osmolality 2 (mOsm/kg)	100	434	174	122	961	N/A	−0.298 ‡
Mean urine osmolality (mOsm/kg)		425	167	132	880	N/A	−0.276 ‡

*n*, sample number; SD, standard deviation; N/A, not applicable. * Paired samples *t* test between BFQ1 and the 24-h urine volume. † Pearson’s correlations between BFQ1 and the 24-h urine volume, significant at *p* < 0.001. ‡ Pearson’s correlations between BFQ1 and the 24-h urine osmolality, significant at *p* < 0.005.

**Table 3:** Reliability of the beverage frequency questionnaire (BFQ).

	*n*	Mean	SD	Minimum	Maximum	*p* *	*R* †
BFQ1 (mL)	121	2797	919	938	5518	0.598	0.799 †
BFQ2 (mL)	60	2746	974	1152	6089		

*n*, sample number; SD, standard deviation. * Paired samples *t* test between BFQ1 and BFQ2. † Pearson’s correlations between BFQ1 and BFQ2, significant at *p* < 0.001.

## Appendix A

The beverage frequency questionnaire (BFQ).

## Appendix B

The visual portion size guide.
